# Transcriptomic Profile of Genes Encoding Proteins Involved in Pathogenesis of Sjögren’s Syndrome Related Xerostomia—Molecular and Clinical Trial

**DOI:** 10.3390/jcm9103299

**Published:** 2020-10-14

**Authors:** Katarzyna Błochowiak, Piotr Celichowski, Bartosz Kempisty, Katarzyna Iwanik, Michał Nowicki

**Affiliations:** 1 Department of Oral Surgery and Periodontology, Poznan University of Medical Sciences, 61-812 Poznan, Poland; 2 Department of Histology and Embryology, Poznan University of Medical Sciences, 61-701 Poznan, Poland; p.celichowski@gmail.com (P.C.); bkempisty@ump.edu.pl (B.K.); mnowicki@ump.edu.pl (M.N.); 3 Department of Anatomy, Poznan University of Medical Sciences, 61-701 Poznan, Poland; 4 Department of Veterinary Surgery, Institute of Veterinary Medicine, Nicolaus Copernicus University in Torun, 87-100 Torun, Poland; 5 Department of Clinical Pathomorphology, Poznan University of Medical Sciences, 60-355 Poznan, Poland; katarzyna.iwanik@ump.edu.pl

**Keywords:** Sjögren’s syndrome, xerostomia, salivary glands, APP

## Abstract

Sjögren’s syndrome (SS) is characterized by xerostomia. We aimed to investigate and compare gene expressions in the labial salivary glands of SS patients with xerostomia SS (sicca) and without xerostomia SS (non-sicca) and of healthy subjects (HS) by means of microarray analysis, and to find genes involved in xerostomia. The study group comprised 11 SS patients (3 SS (sicca) and 8 SS (non-sicca)) and 9 HS. The relative gene expression changes were validated with RT-qPCR in the larger study group. Among the differently expressed genes belonging to the “secretion” ontology group with a fold change >2 and with a *p* value < 0.05, the Transmembrane P24 Trafficking Protein 10 (TMED10), Protein Disulfide Isomerase Family A Member 4 (PDIA4), Calnexin (CANX), Amyloid Beta Precursor Protein (APP), and Transmembrane BAX Inhibitor Motif Containing 6 (TMBIM6) gene expressions in both SS (sicca) and SS (non-sicca) groups were lower than in HS. Significant correlations were observed between TMED10, PDIA4, and CANX gene expression in SS (sicca) patients compared to the controls. There were no differences between the SS (sicca) and SS (non-sicca) study groups in the expression of the aforementioned genes. Results indicate their role in the endoplasmic reticulum system, their overlapping function and the loss of the APP neuroprotective function in xerostomia. It has a multifactorial origin and can be triggered by disturbances to the various signaling pathways in saliva secretion.

## 1. Introduction

Sjögren’s syndrome (SS) is a systemic chronic autoimmune disorder characterized by periductal lymphocytic infiltration of the salivary and lacrimal glands, resulting in decreased secretory function of these glands. Saliva plays a crucial role in lubrication, digestion, antimicrobial activity, calcium-phosphate hemostasis, enamel demineralization and wound healing in the oral cavity. Therefore, the results of insufficient salivary flow include difficulties in speaking and eating, sores in the soft tissue, increased risk of opportunistic infections in the oral cavity and the increased caries rate [1,2]. Although SS can trigger systemic changes, the typical feature of SS is a local predominance of the pathological changes in the exocrine glands, such as salivary glands. These changes are representative for all salivary glands. Moreover, the presence of lymphocytic infiltrations in the labial salivary glands (LSG) and its typical microscopic pattern, termed focal lymphocytic sialadenitis (FLS), is the basis for confirming histological SS and is one of the diagnostic criteria of SS. SS is accompanied by a high but differentially ranged prevalence of xerostomia among SS sufferers. 

Hyposalivation is usually attributed to changes in salivary gland tissue morphology, but there is no direct relationship between the severity of morphological changes in the salivary glands and the occurrence and severity of xerostomia. An unresolved issue in SS pathophysiology is the lack of correlation between salivary flow and tissue damage and severity of salivary glands inflammation. There is a group of SS patients who present relevant symptoms of xerostomia with a relatively low degree of inflammation in salivary glands and a small extent of tissue damage. Furthermore, some SS patients do not progress to the more severe disease state and only present symptoms of decreased salivary flow [3]. These observations suggest that SS related xerostomia could be independent of sialadenitis and may be due to other causes. The first step in finding potential factors responsible for SS related xerostomia is to identify genes involved in various salivation regulation pathways as well as to determine their possible expression changes in SS patients presenting with the objective symptoms of xerostomia. 

Both parameters, decreased salivation and FLS, belong to the diagnostic criteria of SS, but they may be detected separately and independently. The microscopic pattern of SS is representative of both major and minor salivary glands. Therefore, it seems that LSG can be used for detecting the genes involved in SS pathogenesis, as well as the genes associated with xerostomia. Previous studies revealed the differences in the gene expression profile in LSG from SS patients in comparison to healthy control subjects. However, the genes exclusively responsible for hyposalivation in SS have not been determined so far [4,5]. To address this need, it seems valuable to research the genes potentially involved in SS related xerostomia.

It is likely that SS related xerostomia has a multifactorial origin and is associated with changes in the quantity and quality of salivary proteins. Saliva secretion is regulated by various neuronal, endocrine and paracrine mechanisms including stimulation from parasympathetic and sympathetic nervous system, neuropeptides secretion and the locally released inflammatory mediators [6]. Moreover, there is a group of ion channel or transmembrane proteins regulating calcium signaling and homeostasis, muscarinic or α-adrenergic receptors activation and ion transport which are responsible for saliva secretion [6]. The complex system of saliva secretion can be impaired on different levels and by disturbances in the various signaling pathways.

A few potential mechanisms involved in xerostomia in SS patients have been described so far [6]. Some morphological changes and the protein composition found in salivary glands and saliva reflect pathological processes related to SS and concurrent xerostomia such as inflammation, autophagy, immunogenicity, autoimmune reactions and apoptosis. Recent studies have highlighted a direct relationship between intraglandular inflammation and decreased salivation [6]. Salivary glands in SS exhibit chronic inflammation and the levels of pro-inflammatory cytokines are significantly increased in the saliva of SS patients compared to the controls [6]. Pro-inflammatory cytokines alter the tight junction integrity of epithelial cells in salivary glands and cause neuronal damage. The increased level of IL-17 in the lymphocytic infiltrating regions of the salivary glands disturbs the tight junction protein complex through activating the NFκB signaling pathway, decreases water transport, and consequently causes hyposalivation. Moreover, severe intraglandular inflammation impairs blood supply to salivary glands and triggers decreased salivation. The antibodies created in SS act against the muscarinic receptor (M3R) by hindering neural stimulation and can disturb the secretory function of exocrine glands. Cytokine-induced inhibition of neurotransmitters and blockading of M3R by anti-muscarinic autoantibodies could result in xerostomia [6]. Another possible mechanism that induces xerostomia in SS is downregulation of aquaporins (AQP5) and their abnormal distribution in salivary glands. Other possible causes of decreased salivation in SS are severe apoptosis of ductal epithelial cells and Ca^2+^-dependent signaling pathway dysregulation [6]. SS patients presented with decreased expression of some calcium channel proteins are involved in saliva secretion such as stromal interaction molecule-1 (STIM1), Orai channels and inositol 1,4,5 triphosphate (IP3) [3,5,6].

Despite the progress made in recent years in identifying genes involved in the initiation and progression of SS, the causative factors of SS related xerostomia have not been clearly defined. We therefore performed gene expression profiling to understand the mechanisms of SS related xerostomia and identify potential biomarkers for prognosis or diagnosis of this condition. In this study, we aimed to investigate and compare gene expression locally in LSG obtained from SS patients both with and without xerostomia and from healthy subjects by microarray analysis, and also to find genes potentially involved in the development of SS related xerostomia. We performed gene ontology and pathway analysis to define cellular and molecular pathways differentially expressed in SS related xerostomia that may be causally related to the development of the disease. 

## 2. Materials and Methods

### 2.1. Study Groups

The study group comprised 11 patients diagnosed with primary SS. Of these, 3 patients presented xerostomia (SS (sicca)) and 8 did not present xerostomia (SS (non-sicca)). Nine healthy subjects (HS) were included as healthy controls. All samples were analyzed by microarray gene expression profiling. The study group used for validation of the relative changes in gene expression obtained in the microarray analysis comprised 22 SS patients (11 SS (sicca) and 11 SS (non-sicca)) and 9 HS. The SS patients were diagnosed according to the American-European Consensus Group (AECG) criteria of 2016 [7,8,9,10]. Exclusion criteria included the presence of IgG4 syndrome, lymphoma, sarcoidosis, essential mixed cryoglobulinemia or infection by hepatitis B or C virus or human immunodeficiency virus. The microscopic confirmation of SS was based on the presence of FLS with a focus score (FS) ≥1 per 4 mm^2^ of glandular tissue. A lymphocytic focus was defined as a dense aggregate of 50 or more mononuclear cells adjacent to normal-appearing mucous acini in salivary gland lobules that lacked ductal dilatation [11,12]. The presence of FLS and non-specific sialadenitis was excluded in all HS group. Laboratory assessments included anti-Ro/SSA and anti-La/SSB, ANA profile and rheumatoid factor (RF). Ocular and oral dryness were assessed using Schirmer’s test and unstimulated whole salivary flow (USWSF), respectively. Xerostomia was diagnosed based on a positive result in USWSF. Volumes of ≤1.5 mL/15 min in USWSF were marked as abnormal saliva secretion and as a diagnostic criterium of xerostomia [13]. USWSF lasting for 15 min was performed 2 h after a meal using the standardized collection procedure. Saliva was collected in a graduated tube via a funnel every 2 min. All SS (sicca) patients met this criterium of xerostomia. Lacrimation in Schirmer’s test ≤5 mm in one eye was marked as abnormal tear secretion and a positive result of Schirmer’s test [14,15]. 

The study was conducted in accordance with the guidelines of the Declaration of Helsinki. The protocol for this study was approved by the Bioethics Committee of Poznan University of Medical Sciences, Poland (number 403/17). All patients provided signed written informed consent.

### 2.2. Labial Salivary Gland Specimens

Labial salivary gland samples were harvested under local anesthesia from the lower lip from SS patients and HS as described [16]. LSG samples from HS were obtained during surgical removal of the mucocele. Following surgery, some LSG samples were immediately stored in RNA later at −20 °C until they were later processed for molecular studies. Other samples from the same biopsy used for histopathological confirmation of SS and for assessment of FS were fixed in 10% buffered formalin. There was no evidence of inflammation in LSG from the HS.

### 2.3. RNA Extraction

Tissue samples were homogenized using a homogenizer. Total RNA was extracted from the LSG samples using a Universal RNA Purification Kit (EURx cat: E3598). The concentration of total RNA was measured from the optical density at 260 nm, and the RNA purity was determined based on the 260/280 nm absorption ratio (higher than 1.8) (NanoDrop Spectrophotometer, Thermo Fisher Scientific, Waltham, MA, USA). Each sample of RNA was diluted to 100 ng/µL for a subsequent microarray assay, 100 ng of RNA from each sample was taken.

### 2.4. Microarray Expression Analysis 

The Affymetrix procedure and methods of analysis were described previously [17,18,19]. Total RNA (100 ng) from each patient sample was subjected to two round sense cDNA amplification (GeneChip™ 3′ IVT PLUS Reagent Kit). The resulting cDNA was used for biotin labeling and fragmentation by Affymetrix GeneChip^®^ WT Terminal Labeling and Hybridization (Affymetrix, Waltham, Massachusetts, USA). Biotin-labeled fragments of cDNA (5.5 μg) were hybridized to Affymetrix Human Genome U219 Array. The microarrays were then washed and stained according to the technical protocol, using an Affymetrix GeneAtlas Fluidics Station. The array strips were scanned employing a GeneAtlas System Imaging Station. The preliminary analysis of the scanned chips was performed using Affymetrix GeneAtlas™ Operating Software. The quality of gene expression data was checked according to the quality control criteria provided by the software. The CEL files obtained were imported into downstream data analysis software.

All analyses were performed using BioConductor software, based on the statistical R programming language. For background correction, normalization and summation of raw data, the Robust Multiarray Averaging (RMA) algorithm implemented in the “affy” package of the BioConductor was applied. Biological annotation was taken from the BioConductor “hgu219.db” package, where an annotated data frame object was merged with a normalized data set, leading to a complete gene data table. The statistical significance of the genes analyzed was obtained by moderated t-statistics from the empirical Bayes method. The resulting *p* value was corrected for multiple comparisons using Benjamini and Hochberg’s false discovery rate. The selection of significantly changed gene expressions was based on an expression fold change ˃2, *p* < 0.05. 

Functional annotation clustering of differentially expressed genes was performed using DAVID (Database for Annotation, Visualization and Integrated Discovery). Gene symbols for up- or down-regulated genes from each of the groups being compared were loaded to DAVID by a “RDAVIDWebService” BioConductor package. For further analysis, we chose enriched GO terms, which have at least 5 genes and a *p* value (Benjamini) < 0.05. The enriched GO terms were subjected to a hierarchical clusterization algorithm and presented as a heatmap. From the differently expressed genes we selected those with a fold expression ratio in SS (non-sicca) patients <1.5. These genes were subjected to analysis using STRING10 software.

Interactions between proteins coded by genes and genes themselves were investigated by means of STRING10 software (Search Tool for the Retrieval of Interacting Genes) [20,21]. The STRING database contains information on protein/gene interactions, including experimental data, computational prediction methods, and public text collections. The STRING database engine provided us with a molecular interaction network formed between the genes in question. The search criteria are based on co-occurrences of genes/proteins in scientific texts (text mining), co-expression, and experimentally observed interactions.

### 2.5. Quantitative Real-Time PCR Validation

The relative changes in gene expression results obtained from the microarray analysis were validated with the use of a real-time quantitative polymerase chain reaction (RT-qPCR). qPCR was performed by means of the CFX96 Deep Well Real-Time System (BIO_RAD), with a smART RT-qPCR kit (cat. e0806, EURx), according to the manufacturer’s protocol. The primers used for qPCR (Table 1) were designed using Primer 3 software (version 0.4.0, Whitehead Institute for Biomedical Research, Cambridge, MA, USA).

The primers were purchased from the Integrated DNA Technologies, Inc. 1710 Commercial Park, Coralville, Iowa 52241, USA.

The real-time PCR program included a 15-min denaturation step, followed by a three-step amplification program: denaturation at 94 °C for 15 s, annealing at 55 °C for 30 s, and extension at 72 °C for 30 s. The specificity of reaction products was checked by determining the melting points (0.1 °C/s transition rate).

The gene expression was normalized to B2M and B-Act genes with the Pfaffl Ratio method.

## 3. Results

All subjects were of Caucasian origin. The summarized demographic, serological and histological characteristics of the SS (sicca), SS (non-sicca) patients and HS used in microarray analysis are presented in Table 2. 

Whole transcriptome profiling by means of Affymetrix microarray allowed us to analyze the gene expression changes between HS, SS patients with (SS (sicca)) and without xerostomia (SS (non-sicca)). Using Affymetrix Human Genome U219 Array, we examined the expression of 49,387 human transcripts. Genes with a fold change ˃2 and with a corrected *p* value < 0.05 were considered as differentially expressed. This set of genes consisted of 2111 different transcripts in SS (sicca) patients and SS (non-sicca) patients. Subsequently, the genes were used to identify significantly enriched GO BP terms. 

**Figure 1:**
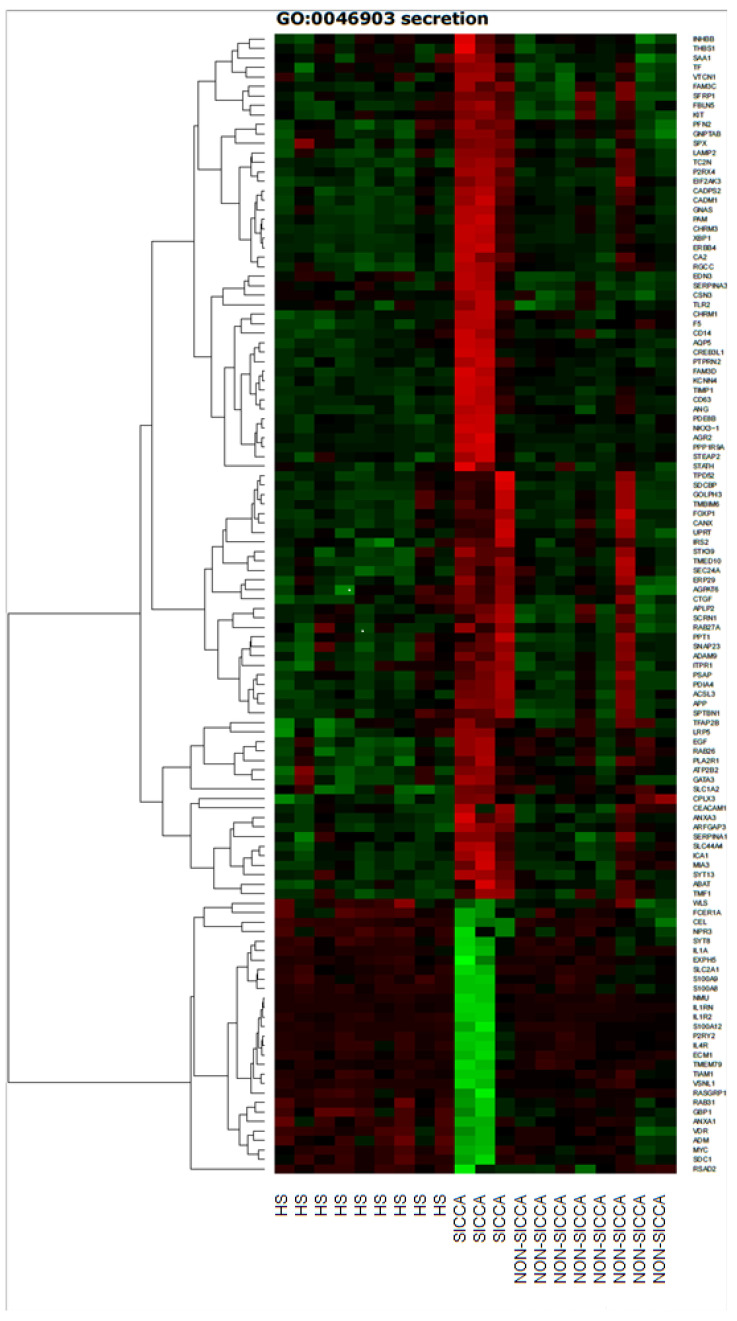
Hierarchical clustering of data from the microarray analysis of gene expression in LSG from patients with SS with (sicca) and without (non-sicca) xerostomia and from the control (HS). Samples with similar patterns of expression of the genes studied will cluster together, as indicated by the dendrogram. Red indicates a higher expression of the gene in SS patients and HS compared to the reference (up-regulated), and green indicates a lower expression of the gene compared to the reference (down-regulation).

DAVID (Database for Annotation, Visualization and Integrated Discovery) software was used to extract the genes belonging to the regulation of “secretion” gene ontology Biological Process term (GO BP). We found that 120 genes from these GO BP terms were significantly represented in down-regulated gene sets. These sets of genes were subjected to a hierarchical clusterization procedure and presented as a heatmap (Figure 1).

The set of the differentially expressed genes belonging to “secretion” GO BP terms with their official gene symbols, fold changes in expression, corrected *p* values and fold change ratio is shown in Table S1.

**Figure 2:**
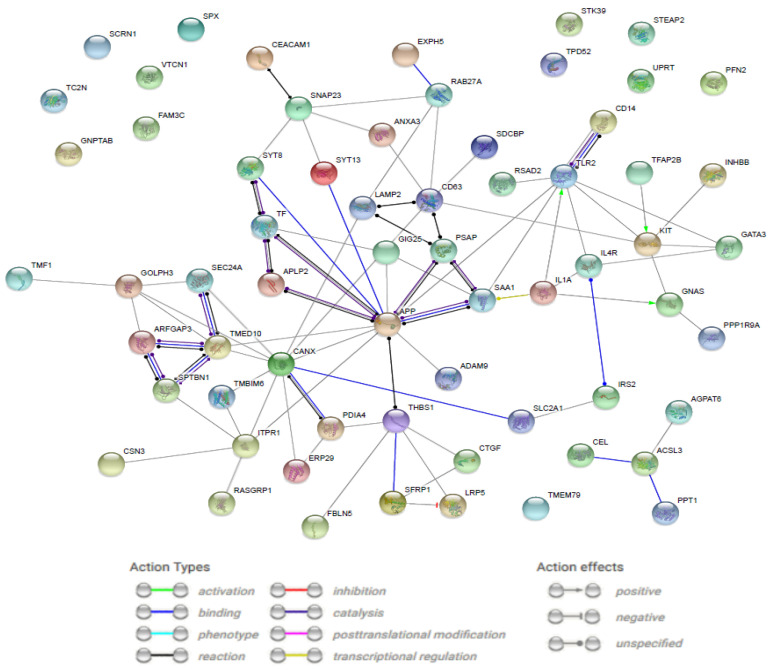
STRING-generated interaction network among differentially expressed genes belonging to the “secretion” ontology group. The intensity of the edges reflects the strength of the interaction score. Prediction methods applied: text mining, co-expression, and experimentally observed interactions.

A STRING-generated interaction network was created with differentially expressed genes belonging to the “secretion” GO BP term. The intensity of the edges reflects the strength of the interaction score (Figure 2). 

From the differently expressed genes that encode secreted proteins (identified using the ‘secretion’ GO BP term), we selected 5 genes: Transmembrane P24 Trafficking Protein 10 (TMED10), Protein Disulfide Isomerase Family A Member 4 (PDIA4), Transmembrane BAX Inhibitor Motif Containing 6 (TMBIM6), Calnexin (CANX) and Amyloid Beta Precursor Protein (APP) and evaluated the microarray results with them. In SS patients both with xerostomia (sicca) and without xerostomia (non-sicca), TMED10, PDIA4, CANX, APP, and TMBIM6 expression was lower than in HS. Statistically significant correlations were observed between TMED10, PDIA4, CANX, APP and TMBIM6 gene expression in patients SS (sicca) compared to the control. There were no differences between the SS (sicca) and SS (non-sicca) study groups in terms of the expression of the aforementioned genes. The results are shown in Table 3.

These genes were subjected to validation with qPCR. The summarized demographic, serological and histological characteristics of the SS (sicca) and SS (non-sicca) study groups used in RT-qPCR are presented in Table 4.

The HS study group used for validating the relative gene changes with RT-qPCR consisted of 9 individuals. The mean age and range in the HS group was 40 (21–60) years and the ratio of Female/Male was 8/1.

**Figure 3:**
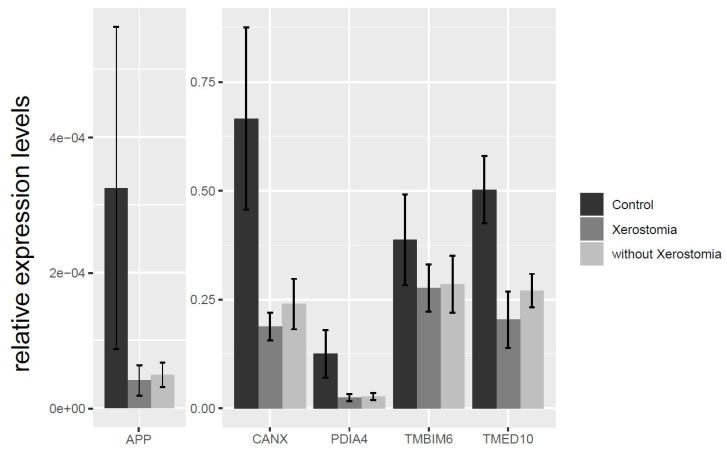
Comparison of APP, TMBIM6, TMED, CANX, and PDIA4 relative gene expression between healthy control subjects (HS), SS patients with xerostomia SS (sicca) and SS patients without xerostomia SS (non-sicca).

From the RT-qPCR, we found that expression of CANX, PDIA4, and TMED10 genes in SS (sicca) was significantly decreased in comparison to HS. No significant correlations were observed in CANX, APP, PDIA4, TMED10 and TMBIM6 gene expression between SS (sicca) and SS (non-sicca) group (Figure 3).

Finally, the TMED10 relative expressions in both SS (sicca) and SS (non-sicca) groups were significantly lower than in the HS group. The results are presented in Table 5.

## 4. Discussion

Although it is clear that there are changes in the expression of certain genes in SS, the exact impact of these genes on the decreased salivary secretion in SS and their relationships remains to be fully elucidated. In the present study, we analyzed genes belonging to secretion ontology group differentially expressed in the salivary glands of SS patients with xerostomia and without xerostomia to determine their potential role in SS related xerostomia. Although we were unable to find any differences in the expression of the genes detected in both SS (sicca) and SS (non-sicca) groups, the expressions of CANX, PDIA4, and TMED10 were significantly decreased in SS (sicca) compared to HS. All the genes explored in our study have an overlapping function and can regulate secretion in SS by a few common mechanisms. This can explain their co-expression and similarly decreased expressions in the same study group. 

A major role in protein homeostasis is played by the endoplasmic reticulum (ER) system, which is responsible for the synthesis, correct folding, modification and transport of proteins and storage of calcium ions. The acinar and ductal cells of the salivary glands are the cells with the most extended ER, especially rough ER (RAR). Furthermore, they are metabolically active cells through the production of saliva, which is rich in proteins. The production and secretion of salivary proteins depends on the ER function and on calcium ions hemostasis Approximately one third of all secretory and membrane proteins is controlled by ER [22,23]. SS related xerostomia has been associated with alterations in quantity and quality of salivary proteins and therefore can stem from disturbances in ER function in salivary glands. Various disorders found in the salivary glands from SS patients, such as intracellular accumulation of mucins, high levels of proinflammatory cytokines, altered posttranslational processing of mucins, dilated ER cisternae and alteration in calcium ions homeostasis, indicate the important impact of ER condition in SS pathology [24,25]. CANX and PDI are lectin-like chaperones that modify and stabilize protein structures in the ER by glycosylation and protein disulfide bonds formation and isomerization. Only modified and properly folded proteins can be transported from the ER through the Golgi apparatus and secretory vesicles and secreted into extracellular environment [26,27]. In our opinion, decreased expression of genes encoding CANX and PDIA4, in SS sufferers with and without xerostomia compared to HS can lead to accumulation of misfolded proteins in the ER and changes in the quantity and quality of salivary proteins. However, despite qualitative differences in the composition of saliva in patients with xerostomia, there are no differences in the expression of genes responsible for protein modifications between SS (sicca) and SS (non-sicca) patients. On the other hand, CANX and PDI can restore ER homeostasis by extensive protein folding. Therefore, their decreased expression can result from the disturbances in cellular response to misfolded or unfolded proteins accumulation in the ER or activation of pro-apoptotic mechanisms, autophagy and protein degradation. It is unclear if decreased expression of the genes is exclusively associated with SS related xerostomia. Xerostomia seems to be more associated with an imbalance between protein groups and encoding genes rather than with specific gene expression changes. Xerostomia occurrence may reflect potentially adaptive or protective mechanisms activation or inhibition in response to common pathological processes related to SS.

Another mutual pathway comprising PDIA4, APP and CANX genes is an ER dependent mechanism observed in both neurodegenerative and autoimmune diseases. Montibeller et al. revealed the activation of cellular response to misfolded or unfolded proteins in the ER known as unfolded protein responses (UPR) and increased expression of UPR related genes in neurodegenerative disorders such as Alzheimer’s disease (AD), amyotrophic lateral sclerosis (ALS) and frontotemporal lobar degeneration (FTLD) [23]. These disorders are characterized by an increased expression of APP and an increased level of β-amyloid peptides, which forms deposits. Contrary to the expression of these genes in neurodegenerative diseases, we reported a decreased expression of APP and the UPR indicative genes. Increased accumulation of unfolded proteins, an impairment of the defense mechanism against them and prolonged UPR activation induce apoptosis and a neuron destruction [28]. ER overload predisposes to neuronal damage and neurodegeneration [23]. ER impairment inducing neuronal damage can explain the neuronal dysregulation of saliva production resulting in decreased salivation in SS-related xerostomia. Saliva secretion is regulated mainly by the parasympathetic nervous system and the function of muscarinic receptors. In both conditions, AD- and SS related xerostomia, there is a loss of cholinergic function. In AD this cholinergic dysfunction leads to cognitive impairments and in SS can result in xerostomia. Furthermore, the M1 muscarinic agonist AF102B known as Cevimeline, previously prescribed in the treatment of dry mouth in SS, can reduce the β-amyloid peptide level and its inhibition as well as the enhanced secretion of APP. This is done by the activation of the M1 receptor with its agonists via α-secretase activation and γ-secretase inhibition [29]. APP plays a neuroprotective role, preventing, in principle, the formation of β-amyloid peptide. The decreased expression of APP in SS sufferers with xerostomia compared to HS can reflect the loss of APP neuroprotective function and the cholinergic deficiency in SS related xerostomia. Furthermore, Hoshino et al. showed that overexpression of ER chaperones, such as CANX and calreticulin (CRT) and glucose-regulated protein 78 (GRP78) in cells, decreased the amount of β-amyloid peptide, which was consistent with previous results [30]. The direct interaction of APP and CANX inhibits the translocation of APP from the ER to the Golgi apparatus, inhibiting its maturation and decreasing β-amyloid peptide levels [30]. This correlation had been studied previously in neurodegenerative diseases [31]. Except for the neuroprotective effect of APP on acetylocholine receptors, another possible impact of APP and its product of proteolysis, β-amyloid peptide, on saliva secretion is associated with calcium transport. The addition of β-amyloid peptide to neuronal cells leads to a rise in the concentration of intracellular Ca^2+^ by stimulating the influx of extracellular Ca^2+^ and efflux of ER Ca^2+^ [30]. 

Another important cause of dry mouth in SS is the imbalance between insults from adrenergic and acetylocholine receptors. Skopouli et al. showed that increased signaling through adrenergic receptors enhanced the production and secretion of salivary proteins, but also activated UPR [32]. 

The activation of UPR induces the production of pro-inflammatory cytokines and induces autophagy as a survival mechanism in response to ER stress, cytosolic stress and inflammation. Autophagy is involved in the removal of potentially toxic cytoplasmatic components and maintenance of cellular homeostasis during stress. TMED10 is a well-known regulator of autophagy. Its function was described previously in neurodegenerative diseases [33]. The expression of TMED10 in AD patients was considerably decreased, and down-regulation of TMED10 increased β-amyloid production. TMED10 depletion is correlated with increased autophagy activation. Our study revealed decreased expression of TMED10 in both SS (sicca) and SS (non-sicca) compared to HS but there were no differences in TMED10 expression between SS (sicca) and SS (non-sicca) groups. These findings indicate that SS related xerostomia does not result from increased activation of autophagy. 

Prolonged UPR activation or an additional stressor disturbs this adaptive mechanism and results in apoptosis [32,34]. TMBIM6 detected in our study is a transmembrane protein, located at the ER, that has a relevant role in preventing apoptosis. It regulates ER calcium homeostasis, impacting stimulated calcium release and calcium-mediated cell death. TMBIM6 negatively modulates ER calcium release and acts as an ani-apoptotic factor under conditions of the accumulation of misfolded proteins in the ER lumen [35,36]. Moreover, TMBIM6 protects the cells from the ER dysfunction, but in our study TMBIM6 expression did not correspond to CANX and PDIA4 expression. Furthermore, its expression was independent of SS related xerostomia.

The limitations of this study arise mainly from the selection of SS patients, the disease duration and the heterogeneity of accompanying symptoms. This limitation impeded our ability to find genes directly responsible for decreased saliva secretion. The proper patient selection allows all possible factors disturbing saliva secretion to be excluded. Another limitation of this study is the difference between the cellularity and gene profile of different cell types within LSG, including periductal, acinar and infiltrated mononuclear cells. In our study, a few patients were taking drugs that reduce saliva secretion. These drugs may decrease secretion and change the objective tests of saliva secretion assessment. Many drugs applied in the therapy of hypertension, depression and other diseases can reduce salivation. Therefore, current and previous therapy and co-existing diseases and therapies might have interfered with our results. 

## 5. Conclusions

There is a group of genes involved in SS pathology and the changes in their expression can be the consequence of pathological processes accompanying SS. SS related xerostomia is a consequence of their multifactorial action. There are many reciprocal interactions between the genes found in LSG, a finding that reflects their similar and overlapping function. Moreover, these genes are involved in various signaling pathways regulating the complex mechanism of saliva secretion. Among the genes changed in SS and responsible for saliva secretion, there is a set of genes involved in ER function and in the loss of cholinergic stimulation. All genes determined in our study play a role in these processes. Although we found no differences between the SS (sicca) and SS (non-sicca) groups and no direct relationship between xerostomia and gene expression, for a comprehensive assessment of SS related xerostomia, the expression of other genes from the “secretion “ontology group should be determined and verified in larger SS patient samples. 

## Figures and Tables

**Table 1:** Sequences of primers used for real-time quantitative polymerase chain reaction (RT-qPCR) analysis.

Gene Name	Forward Primer (5′–3′)	Reverse Primer (5′–3′)
Amyloid Beta Precursor Protein (APP)	ggccctggagaactacatca	aatcacacggaggtgtgtca
Calnexin (CANX)	tgtagcccttcctgtgttcc	ttgactgacagtgccaccat
Protein Disulfide Isomerase Family A Member 4 (PDIA4)	gagagtggggaggatgtcaa	gtcaaaggtctttcccacca
Transmembrane P24 Trafficking Protein 10 (TMED10)	gtggaggcgaaaaattacga	aagaagcgtcgcaggtagaa
Transmembrane BAX Inhibitor Motif Containing 6 (TMBIM6)	ttggatccatttggcttttc	ggctggatggtcacttcatt

**Table 2:** Demographic, serological and histological characteristics of the SS (sicca), SS (non-sicca) patients and HS participating in the microarray analysis.

Parameters	Healthy Subjects (HS)		SS Patients	
			SS (Sicca)	SS (Non-Sicca)
Number of individuals	9	3	8	
Gender, Female/Male, *n*	8/1	3/0	7/1	
Age, mean ± SD years	40.0 ± 18.4	57.3 ± 1.2	48.3 ± 10.1	
Age, median (range), years	33 (21–60)	58 (56–58)	51.5 (35–59)	
Xerophtalmia, *n*	0	1	3	
Focus score ^a^, *n*				
1	0	1	2	
2	0	1	2	
3	0		3	
4	0	1	1	
Ro/SSA antibodies positive, *n*	0	3	6	
La/SSB antibodies positive, *n*	0	1	5	
Antinuclear antibodies positive, *n*	0	2	8	
Rheumatoid factor, *n*	0	1	3	
USWSF, mean ± SD ml/15 min	4.17 ± 1.54	0.7 ± 0.3	3.1 ± 0.9	

*n*, number; %, percentage; ^a^ the number of foci/4 mm^2^ of the tissue; RF, rheumatoid factor; USWSF, unstimulated whole salivary flow; SD, standard deviation.

**Table 3:** Comparison of TMED10, PDIA4, CANX, APP, and TMBIM6 gene expression in SS patients with xerostomia SS (sicca) and without xerostomia SS (non-sicca) and HS.

Gene Symbol	Entrez Gene ID	SS (Non-Sicca) Fold Change	SS (Sicca) Fold Change	SS (Non-Sicca) Adjusted *p* Value	SS (Sicca) Adjusted *p* Value
APP	351	−1.230085	−4.00201	0.9824056	0.00029089
CANX	821	−1.226044	−2.8748997	0.896879335	0.036051034
TMBIM6	7009	−1.230474	−4.248253	0.9453528	0.02556178
TMED10	10972	−1.590254	−2.6071216	0.839470532	0.005202518
PDIA4	9601	−1.341286	−3.9323753	0.922175914	0.000558442

**Table 4:** Demographic, serological and histological characteristics of the SS (sicca) and SS (non−sicca) patients participating in the RT-qPCR analysis.

Parameters	SS (Sicca)	SS (Non-Sicca)
Number of individuals	11	11
Gender, *n*		
Female/Male	9/2	10/1
Age, mean ± SD, years	52.8 ± 11.1	50.9 ± 9.9
Age, median (range), years	56 (34–71)	52 (35–64)
Xerophtalmia, *n* (%)	9 (81.8)	2 (18.18)
Focus score ^a^, *n*		
1	2	4
2	3	2
3	3	3
4	3	2
Ro/SSA antibodies positive, *n*	6	8
La/SSB antibodies positive, *n*	6	2
Antinuclear antibodies positive, *n*	9	8
Rheumatoid factor, *n*	7	4
USWSF, mean ± SD ml/15 min	0.5 ± 0.5	3.4 ± 0.9

*n*, number; %, percentage; ^a^ the number of foci/4 mm^2^ of the tissue; RF, rheumatoid factor; USWSF, unstimulated whole salivary flow; SD, standard deviation.

**Table 5:** APP, TMBIM6, TMED, CANX, and PDIA4 relative gene expression in healthy control subjects (HS), SS patients with xerostomia SS (sicca) and SS patients without xerostomia SS (non-sicca).

	APP	TMBIM6	TMED10	CANX	PDIA4
Kruskal Wallis	0.32	0.54	0.0033	0.03	0.02
Dunn test SS (sicca) vs HS	0.0735	0.3417	0.0008	0.0192	0.0176
Dunn test SS (non-sicca) vs HS	0.2121	0.2618	0.0019	0.0072	
Dunn test SS (sicca) vs SS (non-sicca)	0.1863	0.1352	0.3819	0.3451	0.2502

## Supplementary Materials

The following are available online at https://www.mdpi.com/2077-0383/9/10/3299/s1, Table S1: Fold changes, adjusted *p* values of differentially expressed genes belonging to the “secretion” GO BP term in SS (sicca) and SS (non-sicca) groups.
