# Supplementary material for: Transcriptomic Profile of Genes Encoding Proteins Involved in Pathogenesis of Sjögren’s Syndrome Related Xerostomia—Molecular and Clinical Trial
Source: J Clin Med. 2020 Oct 14;9(10):3299. doi: 10.3390/jcm9103299 (PMC7602267; doi:10.3390/jcm9103299)
Supplement: Supplementary file 1 [file jcm-09-03299-s001.pdf]

Table S1. Fold changes, adjusted *p* values of differentially expressed genes belonging to the "secretion" GO BP term in SS (sicca) and SS (non-sicca) groups.

Gene Symbol	Fold Change SS (Non-Sicca)	Fold Change SS (Sicca)	Adjusted *p*-Value SS (Non-Sicca)	Adjusted *p*-Value SS (Sicca)	Entrez Gene ID
CA2	−2.315699782	−6.780481448	0.790837362	0.0000431	760
AGR2	−2.246870999	−6.632197988	0.979870534	0.000161027	10551
CHRM3	−1.745830276	−5.359431593	0.867931575	0.000023	1131
KCNN4	−1.862417007	−4.702153902	0.912743295	0.0000856	3783
F5	−2.89578281	−4.551424261	0.800548978	0.000265368	2153
ADAM9	−1.316631	−4.313445	0.9192185	0.00441691	8754
CREB3L1	−1.842092563	−4.26435664	0.93021917	0.00082783	90993
XBP1	−1.733284877	−4.256037637	0.878475037	0.0000134	7494
TMBIM6	−1.230474	−4.248253	0.9453528	0.02556178	7009
NKX3-1	−1.959827	−4.147546	0.9568564	0.000868476	4824
SFRP1	1.054841839	−4.137442769	0.984379371	0.043535177	6422
CPLX3	−4.018686	−4.06943	0.6799013	0.01208186	594855
SDCBP	−1.187522	−4.04175	0.9710176	0.02372376	6386
SERPINA3	1.460010446	−4.036824664	0.831872232	0.0072786	12
APP	−1.230085	−4.00201	0.9824056	0.00029089	351
GOLPH3	−1.289845	−3.946811	0.9240716	0.04478896	64083
PDIA4	−1.341286148	−3.932375385	0.922175914	0.000558442	9601
CADM1	−2.019093783	−3.751190594	0.814585037	0.0000212	23705
RGCC	−2.144264702	−3.709992396	0.800548978	0.0000848	28984
THBS1	1.059190388	−3.671840026	0.905319886	0.000704756	7057
FOXP1	−1.548514811	−3.668318821	0.867931575	0.021533834	27086
PTPRN2	−1.528593428	−3.602128512	0.953327265	0.0000232	5799
STATH	−1.5339666	−3.568022611	0.991924898	0.010091618	6779
FAM3D	−1.792252886	−3.566413894	0.872040593	0.0000616	131177
SYT13	−1.212553529	−3.557524421	0.966997456	0.0000669	57586
AQP5	−1.745807307	−3.474188327	0.926753915	0.000263533	362
CADPS2	−1.677603	−3.399037	0.858705	0.000062	93664
CTGF	−1.167188191	−3.301668763	0.982372354	0.028662099	1490
ANG	−2.269629018	−3.236962184	0.846942491	0.0000558	283
ERBB4	−1.713089609	−3.210640076	0.840972522	0.00000892	2066
PAM	−1.553126	−3.187179	0.8527116	0.0000225	5066
EIF2AK3	−1.927130456	−3.185462742	0.839561442	0.000253104	9451
IRS2	−1.284959711	−3.177711575	0.903249384	0.003168011	8660
SLC44A4	−1.970801	−3.150855	0.8889284	0.00016615	80736
ATP2B2	−1.873941	−3.086895	0.8630563	0.007911992	491
P2RX4	−1.50295	−3.016018	0.9454581	0.000145252	5025
PPP1R9A	−1.448428828	−3.012132035	0.995211273	0.000240355	55607
MIA3	−1.842402739	−2.968108581	0.846942491	0.000158271	375056
TPD52	−1.327421	−2.929669	0.9100239	0.01140205	7163
EGF	−2.133689219	−2.884794173	0.73781367	0.001826867	1950
CANX	−1.226043503	−2.874899785	0.896879335	0.036051034	821
TIMP1	−1.63393731	−2.863745884	0.922953864	0.000121115	7076
CSN3	1.309011	−2.830181	0.8626948	0.02782585	1448
TFAP2B	−1.46132528	−2.755497843	0.862694815	0.035472537	7021
PDE8B	−1.702845	−2.719565	0.9111265	0.003005934	8622
CHRM1	−1.847644107	−2.7020044	0.8435801	0.000140967	1128
SLC1A2	−1.95428348	−2.684877893	0.698062015	0.008268875	6506
KIT	1.168524999	−2.609930217	0.89102058	0.0078862	3815
TMED10	−1.590254428	−2.607121685	0.839470532	0.005202518	10972
PSAP	−1.145888	−2.547497	0.9823724	0.008822071	5660

INHBB	1.134489004	−2.515611541	0.900541075	0.018296357	3625
RAB26	−1.939872126	−2.480867998	0.705824843	0.003439846	25837
GATA3	−1.269849309	−2.47250171	0.946700063	0.010374236	2625
VTCN1	1.354908004	−2.468700886	0.800548978	0.005572008	79679
ANXA3	−1.262857	−2.452204	0.8929608	0.0000856	306
CD14	−1.112766	−2.444622	0.9809094	0.000860474	929
ACSL3	−1.262017	−2.387083	0.8740341	0.000554976	2181
STEAP2	−1.418534	−2.379288	0.9871116	0.000753708	261729
GNPTAB	−1.145262	−2.374223	0.9814193	0.002094668	79158
SNAP23	−1.286108	−2.362467	0.952646	0.02434963	8773
STK39	−1.345622151	−2.355844971	0.862694815	0.004807135	27347
LRP5	−1.499841926	−2.321836231	0.863056252	0.018610908	4041
RAB27A	1.013519939	−2.310384054	0.965545111	0.014339841	5873
PLA2R1	−1.668125	−2.295024	0.8311443	0.009190279	22925
PFN2	−1.134314	−2.291889	0.9738305	0.002198721	5217
ARFGAP3	−1.481192892	−2.263934394	0.68332415	0.0000105	26286
SERPINA1	−1.673764402	−2.256128794	0.993499389	0.016246391	5265
AGPAT6	−1.006300472	−2.249632401	0.997569238	0.047394182	137964
EDN3	1.568663173	−2.246532677	0.660591101	0.002440045	1908
APLP2	1.118778	−2.232646	0.9121355	0.01132797	334
TC2N	−1.360208	−2.227485	0.7646354	0.0000155	123036
SAA1	1.446255611	−2.222329225	0.831872232	0.046639694	6288
TF	1.185163	−2.184357	0.8335506	0.004330112	7018
TMF1	−1.347738134	−2.172798624	0.831872232	0.000902922	7110
FAM3C	−1.050636	−2.17207	0.9823724	0.002515679	10447
GNAS	−1.265181689	−2.161799642	0.899626735	0.00018824	2778
SEC24A	−1.431744	−2.140576	0.7781273	0.01573195	10802
SPTBN1	1.050722127	−2.12667403	0.946472831	0.032340493	6711
LAMP2	−1.409126	−2.125203	0.8630563	0.000725617	3920
ITPR1	−1.090888	−2.11527	0.985144	0.01438952	3708
ERP29	−1.210639	−2.11457	0.9053199	0.01141412	10961
SPX	1.164833	−2.092485	0.8409725	0.004650759	80763
PPT1	−1.103776	−2.056105	0.9963049	0.04913632	5538
UPRT	−1.188115	−2.043706	0.8996391	0.02766213	139596
CEACAM1	−1.099703449	−2.041132572	0.924528421	0.0343029	634
ICA1	−1.588196228	−2.030122427	0.862694815	0.0000406	3382
FBLN5	−1.043308	−2.015879	0.9803143	0.003425793	10516
CD63	−1.335474	−2.012781	0.8979428	0.0000307	967
SCRN1	1.002961367	−2.011132387	0.970265404	0.021488791	9805
ABAT	-1.80454	−2.004762	0.800549	0.00073258	18
TLR2	1.073343	−2.00215	0.8630563	0.001607157	7097
SLC2A1	1.434884	2.041067	0.944703	0.003265138	6513
RASGRP1	1.44463	2.045288	0.9193992	0.000525206	10125
VDR	2.115831019	2.073346433	0.789577099	0.003068046	7421
WLS	1.702460585	2.129973321	0.814585037	0.017283908	79971
ADM	1.601144799	2.143069835	0.85643934	0.001791109	133
CEL	1.411035	2.147662	0.800549	0.0000842	1056
ANXA1	1.908474857	2.150081663	0.832930604	0.006278241	301
MYC	2.259848	2.195905	0.8311443	0.005767551	4609
FCER1A	1.851877718	2.202616931	0.814585037	0.00416603	2205
SYT8	1.462028	2.255721	0.8589145	0.000687012	90019
IL1A	1.287214321	2.300114283	0.98177044	0.000607513	3552
RAB31	1.87996	2.331126	0.800549	0.000178105	11031
SDC1	1.930859203	2.332226718	0.862694815	0.002703032	6382
IL4R	1.483682858	2.36419907	0.922995371	0.000112196	3566
EXPH5	1.393668287	2.467395031	0.932434245	0.002996531	23086
TIAM1	1.736981947	2.536660496	0.934358032	0.001675277	7074

GBP1	2.231254085	2.635095619	0.6844031	0.000187975	2633
P2RY2	1.544868	2.885072	0.9048599	0.0000249	5029
S100A12	1.735831998	2.954983075	0.952426113	0.000158271	6283
IL1R2	1.68013362	3.022958371	0.922175914	0.00023231	7850
VSNL1	1.608504852	3.127527026	0.916228964	0.000513257	7447
TMEM79	1.368806608	3.450647133	0.993470113	0.000386272	84283
NPR3	1.721282678	4.055649408	0.774422148	0.000814688	4883
RSAD2	1.345025	4.465685	0.842379	0.007852383	91543
ECM1	1.841249965	4.540495371	0.963827761	0.0000393	1893
NMU	1.912432479	5.338587338	0.95482507	0.000258501	10874
IL1RN	2.043261225	7.10434065	0.999229753	0.000630106	3557
S100A9	3.595271137	7.682950681	0.875843438	0.000558442	6280
S100A8	5.387888	10.71339	0.8713598	0.001466556	6279
